# Positive parenting style and positive health beyond the authoritative: Self, universalism values, and protection against emotional vulnerability from Spanish adolescents and adult children

**DOI:** 10.3389/fpsyg.2022.1066282

**Published:** 2022-12-16

**Authors:** Iria Palacios, Oscar F. Garcia, Marta Alcaide, Fernando Garcia

**Affiliations:** ^1^Department of Developmental and Educational Psychology, University of Valencia, Valencia, Spain; ^2^Department of Methodology, Faculty of Psychology, University of Valencia, Valencia, Spain

**Keywords:** warmth, strictness, parenting styles, self, values, emotional vulnerability

## Abstract

**Introduction:**

Recent research is fully questioning whether the combination of parenting warmth and strictness (the authoritative style) is always identified as positive parenting across the globe. This study analyzes parenting styles and the positive health of adolescents and adult children.

**Methods:**

The sample was 2,090 Spanish children (59.9% women), from four age groups: 600 adolescents, 591 young adults, 507 middle-aged adults, and 392 older adults. Parenting styles (indulgent, authoritative, authoritarian, and neglectful) were obtained by warmth and strictness measures. Children’s positive health was measured by self (family self-concept, self-esteem, and negative self-efficacy), universalism values, and emotional vulnerability.

**Results:**

The main results showed that the indulgent parenting style was associated with equal and even better scores than the authoritative style, whereas the authoritarian and neglectful styles were consistently associated with low scores in positive health indicators for all age groups. However, two triple interactions of sex by age group by parenting style showed that women children from neglectful families reported the lowest family self-concept in old age and the highest emotional vulnerability in middle age.

**Discussion:**

The study findings question the universal benefits of the so-called positive parenting (the authoritative style) for positive health.

## Introduction

The first social nucleus of any person is the family, where children establish their first emotional relational links with their parents (or main caregivers) and where they learn behavioral models adapted to the culture in which they live (Bowlby, 1969; Baumrind, 1978; Darling and Steinberg, 1993; Gimenez-Serrano et al., 2022; Sandoval-Obando et al., 2022). Therefore, parents are the first socializing agent responsible for transmitting the values, beliefs, and attitudes that will shape the personality of adult children (Freud, 1933; Maccoby, 1992; Darling and Steinberg, 1993). In this sense, parental socialization acquires special relevance, understood as the set of strategies and practices that parents use to relate to their children and that will influence their personal and social development (Maccoby, 1992; Darling and Steinberg, 1993). One of the strategies identified by Baumrind (1978) as central to defining the socialization styles of parents was the degree of authority they use in the education of their children, giving rise to three parenting styles: authoritarian, authoritative, and permissive. Baumrind’s model was updated by another approach proposed by Maccoby and Martin (1983) to define parenting styles in two dimensions. Specifically, the so-called two-dimensional model includes the different parental practices based on two independent dimensions (i.e., orthogonal): on the one hand, the warmth dimension, also labeled acceptance/involvement (Lamborn et al., 1991) and responsiveness (Baumrind, 1991), which includes parental practices related to affection and warmth, dialog, the ability to show support to the children, as well as to communicate using inductive reasoning (Darling and Steinberg, 1993; Martinez et al., 2019; Villarejo et al., 2020); and, on the other hand, the dimension of parental strictness, also labeled demandingness, defined by strategies such as the establishment of strict limits and rules, the use of physical and/or verbal coercion, as well as demanding attitudes on the part of the parents (Lamborn et al., 1991; Axpe et al., 2019; Martínez et al., 2021). Based on the combination of these two dimensions, the four parental styles are defined as following: The indulgent style (parents characterized by high warmth and low strictness), the authoritative style (parents characterized by high warmth and strictness), the authoritarian style (parents characterized by low warmth and high strictness), and the neglectful style (parents characterized by low warmth and low strictness) (Lamborn et al., 1991; Darling and Steinberg, 1993; Garcia and Gracia, 2009; Garcia et al., 2019).

Interest in socialization styles emerged in the first half of the last century, and there has been a great deal of research on this subject, especially due to the relationship that has been observed between parenting styles and the degree of child competence and psychosocial adjustment (Baldwin, 1948; Baumrind, 1966; Steinberg et al., 1994; Garcia and Gracia, 2010; Lavriè and Naterer, 2020). Overall, the research findings revealed the relation between parenting styles and differences in child competence and psychosocial adjustment. According to classical research, mostly from studies with European-American families, the authoritative style (i.e., warmth and strictness) has been pointed out as the best because children raised by warm and strict parents obtained better results in the different variables of personal and social adjustment studied in comparison with their peers from the other families (Baumrind, 1966; Steinberg et al., 1989, 1992; Lamborn et al., 1991; Darling and Steinberg, 1993). However, the benefits of the authoritative style, mainly found in studies with white middle-class European-American families, have not always been identified in all cultural contexts across the globe (Pinquart and Kauser, 2018). The discrepant research findings about which parenting is the best to promote child and adolescent development could be explained by the influence of the cultural context in which parents raise their children since families develop and evolve within a social system with values and beliefs specific to each culture (Bronfenbrenner, 1986; Rudy and Grusec, 2001; Garcia and Gracia, 2009), so that, by identifying with these values inherent to their culture, it will be more likely that a certain socialization style will be related to better results in the adjustment variables of the children (Bronfenbrenner, 1986; Darling and Steinberg, 1993; Martínez and Garcia, 2007). Thus, for example, some cross-cultural research conducted with ethnic minorities in the United States, such as African Americans (Baumrind, 1972; Deater-Deckard et al., 1996) and Asian-Americans (Chao, 1994, 2001), showed that the authoritarian style (i.e., strictness without warmth) has been positively related to some psychosocial adjustment variables (Pinquart and Kauser, 2018). The same has been observed in studies conducted with the Arab population, where authoritarian style is the one related to better mental health (Pinquart and Kauser, 2018).

Additionally, in more recent research carried out mainly in European and South American countries (Calafat et al., 2014; Garcia et al., 2019; Martinez et al., 2020), it has been found that indulgent parenting (i.e., warmth without strictness) stands out as the best, being related to equal and even better scores than the authoritative style (Martínez and Garcia, 2008; Garcia and Gracia, 2010; Alonso-Geta, 2012), while children of authoritarian and neglectful parents present a lower level of psychosocial adjustment (Alonso-Geta, 2012; Suárez-Relinque et al., 2019; Gimenez-Serrano et al., 2021). For example, children with indulgent parents reported the highest scores in adjustment indicators such as school adjustment (Fuentes et al., 2015), empathy (Martinez-Escudero et al., 2020), emotional stability (Garcia and Serra, 2019), and showing a higher priority toward social values (Garcia and Serra, 2019; Martinez et al., 2020). In the same way, parenting characterized by warmth without strictness (i.e., the indulgent style) has also been related to lower substance use (Riquelme et al., 2018; Garcia et al., 2020b) and criminal behaviors (Martínez et al., 2013).

### The present study

Parental socialization is a process through which parents transmit to their children the values, beliefs, and learning necessary to function adaptively in the family and cultural context in which they live and ends when the children reach adult age. Overall, differences in adjustment during adolescence increase compared to childhood (Veiga et al., 2015, 2021; Musitu-Ferrer et al., 2019). In this sense, most of the research conducted on the appropriateness of parenting styles has been carried out during the socialization period of itself, that is, with minor children and, above all, in the adolescent stage (Lamborn et al., 1991; Steinberg et al., 1992, 1994; Garcia and Gracia, 2010; Alonso-Geta, 2012; Martínez et al., 2013). However, socialization style can also influence the level of competence and adjustment of children even when they are adults, although little is known about the relations with adulthood (Buri, 1991; Garcia O. F. et al., 2018; Garcia et al., 2021; Candel, 2022; Koutra et al., 2022).

During the socialization process, the child develops a sense of self, which originates from his or her accumulated experiences in different contexts (Shavelson et al., 1976; Marsh and Shavelson, 1985; Harter, 1988), among others, the family (Harter, 1988; Martínez et al., 2021). The child’s self-perceptions as a valuable person and loved by his or her family (i.e., family self-concept) (Garcia F. et al., 2018; Chen et al., 2020; Martínez et al., 2021) are also key in the development of a positive general valuation as a valuable person with qualities (i.e., self-esteem) (Byrne and Shavelson, 1996; Bracken et al., 2000; Martín-Albo et al., 2007). Particularly for personal and good social functioning as a productive member of society, self-efficacy should be developed, that is, the individual belief in the ability to be able to do things well that help the child and adolescent to have achievement and success (Bandura, 1977; Chen et al., 2004). On the opposite side, poor self-efficacy (i.e., negative self-efficacy) is related to greater problems and lower adjustment (Martin et al., 1996; Bandura and Locke, 2003; Ali et al., 2015). In addition, the child in turn must internalize social values (Darling and Steinberg, 1993; Rudy and Grusec, 2001), although the transmission of these values (e.g., by parents or school) does not guarantee that all of them will internalize them (Rudy and Grusec, 2001; Martínez and Garcia, 2007; Garcia et al., 2019). In particular, the social values of self-transcendence and self-conservation are key to producing adjustment behaviors (e.g., prosocial behaviors) and protection against deviant behaviors (Rudy and Grusec, 2001; Martínez and Garcia, 2007; Garcia et al., 2019). Likewise, adjustment can be seriously affected by emotional vulnerability (Steinberg, 2001; Steinberg and Morris, 2001; Riquelme et al., 2018). Thus, processing of situations as dangerous or threatening to oneself can seriously alter personal and social functioning (MacLeod et al., 2002; Mathews and MacLeod, 2005).

Parental socialization ends when the child reaches adulthood (Baumrind, 1978; Maccoby, 1992; Garcia and Serra, 2019), and development continues throughout adulthood and old age. There are differences in adjustment over time for men and women. For example, in self-esteem, men tend to have higher scores than women, whereas, over time, levels decline during adolescence, gradually increase throughout adulthood, and decline sharply in old age (Robins and Trzesniewski, 2005). Therefore, an important question is whether differences in adjustment during adolescence, but also in adulthood, may be related to parenting styles and to which parenting is the most positive. Specifically, whether differences in men and women adjustment at a given time (e.g., adolescence or midlife) can be related to parental socialization and whether these differences follow the same pattern when parenting styles are sorted to identify beneficial and detrimental parenting styles.

Overall, the so-called positive parenting or effective parenting is usually identified as authoritative parenting: The combination of parental responsiveness and demandingness is consistently related to adolescent adjustment, school performance, and psychosocial maturity (e.g., Steinberg and Morris, 2001, p. 88). Positive parenting is defined in terms of greater positive health and lower deviance. According to research mostly conducted with European-American families, only the parental strategy based on greater responsiveness and higher demandingness always benefit children and adolescent in their positive health not only by offering them protection against deviance but also good adjustment and wellbeing (Baumrind, 1966; Steinberg et al., 1989, 1992; Lamborn et al., 1991; Darling and Steinberg, 1993). Specifically, in terms of adjustment and wellbeing, adolescents from warm and strict families reported greater self-conceptions and maturity, while similar benefits are reported by those from other warm families, but without the strictness component (i.e., the indulgent). On the opposite side, adolescents from families characterized by a lack of warmth (i.e., the authoritarian and neglectful) reported the poorest scores. In terms of deviance, again, adolescents from strict families with warm households reported the lowest rates, while similar benefits are obtained by those from other strict families (i.e., the authoritarian). On the opposite side, adolescents from families characterized by a lack of strictness reported the highest scores. However, the so-called positive parenting or effective parenting has been proposed as having universal benefits (Steinberg, 2001), but recent research suggests that not always authoritative parenting can be the best for positive health (Pinquart and Kauser, 2018; Garcia et al., 2019).

This study analyzes the relationship of parental socialization styles with self (family self-concept, self-esteem, and negative self-efficacy), internalization of universalism values, and emotional vulnerability, and using a sample of adolescents and adult children (young, middle-aged, and older). The new previous evidence seriously doubts that the so-called positive parenting (i.e., the authoritative) would be always the best (Pinquart and Kauser, 2018; Garcia et al., 2019). Based on recent research, it can be expected that the indulgent style will be the best adjustment, being related to scores equal to, and even better than, those obtained by the children of authoritative parents in self (family self-concept, self-esteem, and negative self-efficacy), universalism values, and emotional vulnerability.

## Materials and methods

### Sample and procedure

The sample was 2,090 participants, 1,251 womens (59.9%), and 839 mens (40.1%), adolescents and adults (*M* = 36.14 and *SD* = 20.40), with four age groups: adolescents (*n* = 600, 361 womens, 60.2%), aged 12–18 years (*M* = 16.68 and *SD* = 1.59); young adults (*n* = 591, 349 womens, 59.1%), aged 19–35 years (*M* = 23.71 and *SD* = 3.78); middle-aged adults (*n* = 507, 327 womens, 64.5%), aged 36–59 years (*M* = 48.34 and *SD* = 6.37); and older adults (*n* = 392, 214 womens, 54.6%), aged 60 years or older (*M* = 68.91 and *SD* = 7.85). With *a priori* power analysis, a minimum required sample of 1,724 participants was needed to detect, with a power of 0.95 (α = 0.05; 1 − β = 0.95), a small effect size (*f* = 0.10) for the univariate *F*-test among the four parenting styles (Cohen, 1977; Gracia et al., 1995; Pérez et al., 1999; Faul et al., 2009). With the study sample of 2,090 participants, nevertheless, a sensitivity power analysis between the four parenting styles guaranteed the detection of an effect size of at least 0.091 (*f* = 0.091, α = 0.05, 1 − β = 0.95) (Calafat et al., 2014; Fuentes et al., 2015; Queiroz et al., 2020). The responses were collected by online questionnaires with mandatory responses hosted on the University website. Participants generally required about 60 min to respond. Adolescents were recruited from the full list of high schools. Six schools were selected, and one head declined to participate. Young adults were recruited from undergraduate courses. Middle-aged adults were recruited from middle-class neighborhoods. Older adults were recruited by random selection from the full list of senior centers. Four urban senior centers were selected. All participants in this study, including adolescent children and adult children: (a) completed the same questionnaires; (b) they were Spanish, as well as their parents and grandparents; (c) participated voluntarily; (d) informed consent was required, and in the case of adolescents, parental consent was also requested; and (e) the anonymity of the responses was guaranteed.

### Measures

#### Parental socialization

The two main dimensions of parental socialization were measured to define the four parenting styles: warmth and strictness (Steinberg et al., 1992, 1994; Chao, 2001; Alonso-Geta, 2012; Calafat et al., 2014; Garcia et al., 2019, 2020b). The warmth dimension was measured with the 20 items of the Warmth/Affection Scale of the Parental Acceptance-Rejection Questionnaire (PARQ) (Rohner, 2005; Calafat et al., 2014; Garcia O. F. et al., 2018). Warmth items measure certain behaviors concerning affection, care, empathy, and positive parenting in general, as perceived by the children during the adolescent period (e.g., “Say nice things about me,” “Make it easy for me to tell them things that are important to me”). For adult children, the same scale was used but with reference to the past (e.g., “Said nice things about me,” “ They believe in having a lot of rules and sticking to them”). High scores indicate a high degree of warmth. The alpha value was 0.946. Additionally, the strictness dimension was captured with 13 items of the Parental Control Scale of the PARQ (Rohner, 2005; Calafat et al., 2014). Control items assess questions related to the degree of parental supervision and strictness perceived by the children (e.g., “Are always telling me how I should behave,” “They believe in having a lot of rules and sticking to them”). For adult participants, the same scale was used (Senese et al., 2016) but referring to the period of their adolescence (e.g., “Were always telling me how I should behave,” “They believed in having a lot of rules and sticking to them”). Higher scores indicate a greater degree of parental strictness. The alpha value was 0.906. The response scale used for both dimensions was Likert-type with a range from 1 (almost never true) to 4 (almost always true). Based on these two dimensions, the four parenting socialization styles were defined by dichotomizing the sample by the median (50th percentile) of the scores of both dimensions, considering them jointly (Chao, 2001; Calafat et al., 2014; Garcia et al., 2020a). Parenting styles were typified as follows: Indulgent families are those that score above the median in the warmth dimension and below in strictness; authoritative families are those that score above the median in both dimensions (warmth and strictness); authoritarian families are those that score above on strictness and below on warmth; and neglectful families score below on both dimensions (Lamborn et al., 1991; Steinberg et al., 1994; Chao, 2001; Calafat et al., 2014). As has been done in classic and recent studies on parenting styles, the median dichotomization procedure (rather than using predetermined cutoff points) was used. This procedure provides a sample-specific categorization of families (Lamborn et al., 1991; Garcia and Serra, 2019; Queiroz et al., 2020). For example, families in the “neglectful” household are, in fact, relatively more neglectful (i.e., less warm and strict) than the other parents in the sample, but these families we have labeled “neglectful” might be considered to be another family type in a different sample (see Lamborn et al., 1991, p. 1,053).

#### Self

Family self-concept was measured with the 6 items of the Form-5 Self-Concept questionnaire (Garcia and Musitu, 1999), whose Likert-type response scale ranges from 1 (totally disagree) to 99 (totally agree). The person’s self-image within the family system is evaluated in terms of involvement and integration (e.g., “I am happy at home”), so high scores reflect a high family self-concept. The alpha value was 0.806. The AF5 is a widely applied questionnaire for adolescents (Garcia and Serra, 2019; Fuentes et al., 2020; Queiroz et al., 2020) and adults (Garcia et al., 2011; Martinez-Escudero et al., 2020; Villarejo et al., 2020) in the Spanish language. The dimensional structure of this instrument has been tested in many empirical studies with exploratory factor analyses (Garcia and Musitu, 1999) and confirmatory factor analyses (Tomás and Oliver, 2004; Fuentes et al., 2011; Murgui et al., 2012) in different cultural contexts, such as Spain (Murgui et al., 2012), Portugal (Garcia et al., 2006), Brazil (Garcia F. et al., 2018), Chile (Garcia et al., 2011), United States (Garcia et al., 2013), and China (Chen et al., 2020). In addition, its invariance for sex and age has been confirmed in several studies across different languages, such as Brazilian-Portuguese (Garcia F. et al., 2018), Portuguese (Garcia et al., 2006), Chinese (Chen et al., 2020), English (Garcia et al., 2013), and Spanish (Tomás and Oliver, 2004; Fuentes et al., 2011; Murgui et al., 2012). Furthermore, the AF5 has been used to validate other self-concept measures (Garaigordobil and Pérez, 2007; Martín-Albo et al., 2007; Fuentes et al., 2020).

Self-esteem was measured with the Rosenberg Self-Esteem Scale (Rosenberg et al., 1995; Martín-Albo et al., 2007), which consists of 10 items aimed at evaluating feelings of self-respect and acceptance (e.g., “On the whole, I am satisfied with myself”), with a Likert-type response scale ranging from 1 (strongly disagree) to 4 (strongly agree). High scores suggest a high sense of self-esteem. The alpha value was 0.849.

Negative self-efficacy was measured with the six items of the Personality Assessment Questionnaire (PAQ) (Rohner, 1978), whose Likert-type response scale ranges from 1 (almost never true) to 4 (almost always true). This scale allows assessing the person’s perception of his or her ability to perform tasks (e.g., “I feel I am a success in the things I do”). High scores indicate a high degree of negative self-efficacy. The alpha value was 0.719.

#### Universalism values

Universalism values were measured with the eight items of the *Schwartz Value Inventory* (SVI) (Schwartz, 1992), assessing the degree of orientation toward values related to the wellbeing of people and the environment (e.g., “Equality [Brotherhood, equal opportunity for everybody]”). The Likert-type response scale ranges from 1 (not at all important in my life) to 99 (essential in my life). High scores reflect a high priority toward universalism values. The alpha value was 0.826.

#### Emotional vulnerability

Emotional vulnerability was measured with the eight items of the nervousness scale of the psychosocial maturity questionnaire (Greenberger et al., 1975; Garcia and Serra, 2019). This dimension refers to the degree of emotional stability of the person in terms of anxiety and/or tension (e.g., “My mood changes easily,” “I am usually tense, nervous, and anxious”). The Likert-type response scale ranges from 1 (strongly disagree) to 5 (strongly agree). High scores indicate a high degree of emotional vulnerability. The alpha value was 0.779.

### Plan of analysis

To analyze the relationship between parenting styles and children outcomes, a multivariate factorial design (MANOVA) (4 × 2 × 4) was applied between the adjustment variables, which were self (family self-concept, self-esteem, and negative self-efficacy), universalism values, and protection against emotional vulnerability, and the independent variables, which were parenting styles (indulgent, authoritative, authoritarian, and neglectful), sex (women and men), and age (adolescents, 12–18 years; young adults, 19–35 years; middle-aged adults, 36–59 years; and older adults, 60 years and older), to test for possible interaction effects. Subsequently, for those variables that were statistically significant, several analyses of variance (ANOVA) were performed and, finally, for those univariate results that were statistically significant, Bonferroni *post hoc* test (α = 0.05) was applied to compare all possible pairs of means.

## Results

### Parenting styles

The distribution of the participants in the four parental socialization styles is shown in Table 1. The indulgent style consisted of 592 subjects (28.3%), with high scores in warmth (*M* = 73.46 and *SD* = 4.56) and low scores in strictness (*M* = 28.02 and *SD* = 5.58). The authoritative style grouped 456 participants (21.8%), with high scores in warmth (*M* = 72.45 and *SD* = 4.40) and strictness (*M* = 39.81 and *SD* = 5.06). The authoritarian style, with 611 participants (29.2%), scored low in warmth (*M* = 54.81 and *SD* = 10.05) and high in strictness (*M* = 41.89 and *SD* = 5.67). Finally, the neglectful style consisted of 431 participants (20.6%), with low scores in warmth (*M* = 57.11 and *SD* = 9.37) and strictness (*M* = 28.25 and *SD* = 5.70).

**TABLE 1:** Distribution of participants by parenting style, mean scores, and standard deviations for the main parenting dimensions.

	Total	Neglectful	Indulgent	Authoritarian	Authoritative
Frequency	2,090	431	592	611	456
Percentage	100	20.60	28.30	29.20	21.80
Warmth					
*M*	64.42	57.11	73.46	54.81	72.45
*SD*	11.53	9.37	4.56	10.05	4.40
Strictness					
*M*	34.69	28.25	28.02	41.89	39.81
*SD*	8.52	5.70	5.58	5.67	5.06

M, mean; SD, standard deviation.

### Multivariate statistical analysis

Statistically significant differences were obtained for the main effects of parenting style [Λ = 0.754, *F*_(15,5670.6)_ = 40.68, *p* < 0.001], sex [Λ = 0.952, *F*_(5,2054.0)_ = 20.57, *p* < 0.001], and age groups [Λ = 0.931, *F*_(15,5670.6)_ = 9.88, *p* < 0.001], and for the interaction effects between parenting styles, sex, and age groups [Λ = 0.964, *F*_(45,9191.1)_ = 1.66, *p* = 0.004] (Table 2).

**TABLE 2:** MANOVA (4^a^ × 2^b^ × 4^c^) for self (family self-concept, self-esteem, and negative self-adequacy), universalism values, and emotional vulnerability.

Sources of variation	Λ	*F*	*df*_between_	*df*_error_	*p*
(A) Parenting styles^a1–a4^	0.754	40.68	15.00	5670.6	<0.001
(B) Sex^b1,b2^	0.952	20.57	5.00	2054.0	<0.001
(C) Age groups^c1–c4^	0.931	9.88	15.00	5670.6	<0.001
A × B	0.987	1.79	15.00	5670.6	0.030
A × C	0.956	2.08	45.00	9191.1	<0.001
B × C	0.982	2.47	15.00	5670.6	0.001
A × B × C	0.964	1.66	45.00	9191.1	0.004

a_1_, neglectful; a_2_, indulgent; a_3_, authoritarian; a_4_, authoritative.

b_1_, women participants; b_2_, men participants.

c_1_, adolescents (12–18 years); c_2_, young adults (19–35 years); c_3_, middle-aged (36–59 years); c_4_, older (60 years and older).

### Parental socialization styles

In the relationship of parenting styles with the different outcomes, it was observed that the indulgent style was related to the same, and even better scores, than the authoritative style, while the lowest scores corresponded to the authoritarian and neglectful styles (see Table 3).

**TABLE 3:** Means, standard deviations, univariate *F*-values, and Bonferroni’s *post-hoc* analysis of parenting styles for self (family self-concept, self-esteem, and negative self-adequacy), universalism values, and emotional vulnerability by parenting styles.

	Parenting style					
Child outcomes	Neglectful	Indulgent	Authoritarian	Authoritative	*F*_(3,2058)_	*p*
Family self-concept	7.44^c^ (1.49)	8.69^a^ (0.97)	7.08^d^ (1.55)	8.46^b^ (1.04)	191.85	<0.001
Self-esteem	3.16^b^ (0.47)	3.36^a^ (0.44)	3.10^b^ (0.48)	3.35^a^ (0.40)	46.44	<0.001
Negative self-efficacy	1.89^a^ (0.52)	1.58^c^ (0.43)	1.89^a^ (0.52)	1.68^b^ (0.44)	55.32	<0.001
Universalism values	7.44^b^ (1.34)	7.82^a^ (1.26)	7.25^b^ (1.47)	7.73^a^ (1.22)	26.25	<0.001
Emotional vulnerability	2.52^a^ (0.63)	2.20^b^ (0.62)	2.56^a^ (0.63)	2.28^b^ (0.61)	39.87	<0.001

Bonferroni test α = 0.05; a > b > c > d.

#### Self

In family self-concept, the highest scores corresponded to indulgent parenting while the lowest corresponded to authoritarian parenting. Authoritative parenting was related to lower scores than the indulgent, but greater than the neglectful. Within parenting associated with lower scores in family self-concept, the neglectful was related to greater rates compared to the authoritarian. In self-esteem, children from warm families (i.e., indulgent and authoritative) reported greater scores than their peers from authoritarian and neglectful homes. In negative self-efficacy, only children from indulgent families reported the lowest scores, the highest corresponded to those from authoritarian and neglectful households, and those with authoritative parents scored in a middle position.

#### Universalism values

The scores in universalism values showed that indulgent and authoritative were associated with greater scores than authoritarian and neglectful parenting.

#### Emotional vulnerability

The scores in emotional vulnerability showed that again parenting based on warmth (i.e., the indulgent and authoritative) was related to lower scores than non-warmth parenting (i.e., the authoritarian and neglectful).

### Sex and age group

Statistically significant sex differences were found in the five psychosocial adjustment variables evaluated (see Table 4). It was observed that women participants obtained higher scores in family self-concept, negative self-efficacy, emotional vulnerability, and universalism, while men participants reported greater self-esteem.

**TABLE 4:** Means, standard deviations, and univariate *F*-values for self (family self-concept, self-esteem, and negative self-adequacy), universalism values, and emotional vulnerability by children sex.

	Sex			
	Womens	Mens	*F*_(1,2058)_	*p*
Family self-concept	8.02 (1.46)	7.76 (1.46)	15.22	<0.001
Self-esteem	3.19 (0.47)	3.31 (0.50)	38.63	<0.001
Negative self-efficacy	1.79 (0.50)	1.71 (0.50)	18.43	<0.001
Universalism values	7.67 (1.33)	7.38 (1.37)	22.09	<0.001
Emotional vulnerability	2.44 (0.66)	2.32 (0.61)	20.21	<0.001

Bonferroni test α = 0.05.

In addition, statistically significant differences in age were also found in the five measured child outcomes (see Table 5). In family self-concept, all age groups scored higher than older adults, and young adults higher than middle-aged adults. On self-esteem, adolescents scored lower than young and middle-aged adults. On negative self-efficacy, adolescents and older adults scored higher than young and middle-aged adults. On the priority given to values related to universalism, young adults scored higher than adolescents and older adults.

**TABLE 5:** Means, standard deviations, univariate *F*-values, and Bonferroni’s *post-hoc* analysis for self (family self-concept, self-esteem, and negative self-adequacy), universalism values, and emotional vulnerability by age groups.

	Age groups					
Child outcomes	Adolescents	Young adults	Middle aged	Older adults	*F*_(3,2058)_	*p*
Family self-concept	7.95^1^ (1.53)	8.13^a^ (1.40)	7.86^b^ (1.41)	7.60^c,2^ (1.46)	15.92	<0.001
Self-esteem	3.14^b^ (0.48)	3.26^a^ (0.50)	3.32^a^ (0.41)	3.25^a^ (0.43)	14.12	<0.001
Negative self-efficacy	1.83^a^ (0.51)	1.72^b^ (0.50)	1.66^b^ (0.46)	1.83^a^ (0.51)	16.66	<0.001
Universalism values	7.46^b^ (1.42)	7.71^a,1^ (1.23)	7.62^1^ (1.31)	7.38^2^ (1.44)	6.35	<0.001
Emotional vulnerability	2.44 (0.64)	2.41 (0.65)	2.33 (0.64)	2.36 (0.63)	2.51	0.057

Bonferroni test α = 0.05; a > b > c; 1 > 2.

### Interaction effects

**FIGURE 1:**
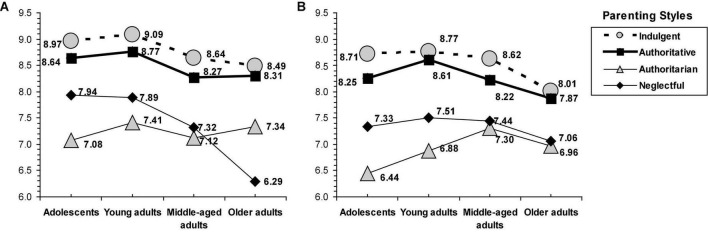
Interaction of parenting styles by age group and sex on family self-concept. **(A)** Family self-concept (women). **(B)** Family self-concept (men).

**FIGURE 2:**
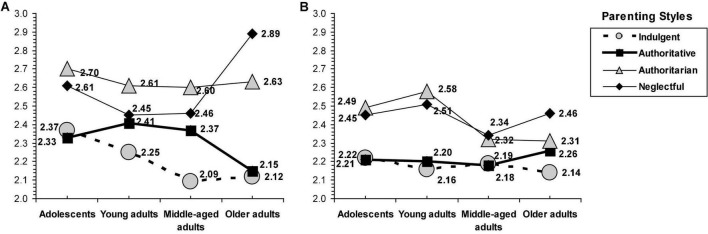
Interaction of parenting styles by age group and sex on emotional vulnerability. **(A)** Emotional vulnerability (women). **(B)** Emotional vulnerability (men).

An interaction effect was observed between parenting style, sex, and age group on family self-concept [*F*_(9,2058)_ = 2.25, *p* = 0.017] (see Figure 1) and emotional vulnerability [*F*_(9,2058)_ = 1.95, *p* = 0.041] (see Figure 2).

In family self-concept, a common pattern by sex was found across all the age groups. Overall, indulgent parenting was related to slightly greater scores than authoritative parenting, while the lower scores corresponded to parenting characterized by a lack of warmth (i.e., neglectful and authoritarian). Interestingly, some variations in the family self-concept were found in non-warmth families (i.e., neglectful and authoritarian), particularly since middle age. Neglectful parenting was related to greater scores than the authoritarian among adolescents and young adults, whereas the scores tended to be equal in the middle-aged groups. However, for older adults women participants, those raised by authoritarian parents scored greater than their peers from neglectful families, whereas for older adults men participants, the scores between neglectful and authoritarian parenting tended to be equal (see Figure 1).

In emotional vulnerability, for women participants, in the group of adolescents, those from indulgent and authoritative families had lower emotional vulnerability than their peers from authoritarian and neglectful homes; in the group of young and middle-aged adults, the lowest scores corresponded to those raised by indulgent parents, the highest to those raised by authoritarian parents, and in a middle position were those from authoritative and neglectful families; finally, in the group of older adults, the lowest scores corresponded by those raised by warm parents (i.e., indulgent and authoritative), the highest by those raised by neglectful families, while those from authoritarian families scored lower than their peers from neglectful households but higher than those from authoritative and indulgent homes (see Figure 2).

In emotional vulnerability, for men participants, the pattern was quite similar for adolescents and young and middle-aged adults: The indulgent and authoritative parenting were related to lower scores compared to the authoritarian and neglectful (the differences between the four parenting styles were lower for middle-aged adults). Similarly, in older adults, the lowest scores corresponded to indulgent parenting, the highest to neglectful parenting, and in a middle position were the authoritarian and authoritative parenting (see Figure 2).

## Discussion

This study analyzes the relationship between parental socialization and positive health in adolescent children but also in adult children (young, middle-aged, and older adults) from a European country (Spain), using the classical two-dimensional socialization model (i.e., warmth and strictness) and four parenting styles (i.e., the authoritative, the indulgent, the authoritarian, and the neglectful). To evaluate positive health, self (family self-concept, self-esteem, and negative self-efficacy), universalism values, and emotional vulnerability were measured. In general terms, the results indicate that indulgent parenting is related to best scores: high in family self-concept, self-esteem, and internalization of values of universalism, and low in negative self-efficacy and emotional vulnerability. Therefore, children from indulgent families (i.e., warmth without strictness), compared to those from authoritative families (i.e., warmth combined with strictness), obtain equal or even better scores in positive health, whereas children from authoritarian and neglectful families obtain scores that are related to more detrimental scores: low scores in family self-concept, self-esteem, and universalism, and high scores in negative self-efficacy and emotional vulnerability.

Overall, the results from the present study about the benefits of indulgent parenting agree with the research carried out in southern European and South American countries (Martínez and Garcia, 2008; Garcia and Gracia, 2010, 2014; Martinez et al., 2020), in which it has been identified that the indulgent style, characterized by high warmth and low strictness, is the best for the adequate adjustment (Garcia et al., 2021). In this sense, in line with the results of this study, the children of indulgent families obtain the best scores in different indicators such as school adjustment (Fuentes et al., 2015), empathy (Martinez-Escudero et al., 2020), emotional stability (Garcia and Serra, 2019), or higher priority toward social values (Martinez et al., 2020). In view of the above, one of the contributions of the present study to the recent scientific literature on parental socialization and children’s adjustment is that the use of parental practices based on affection and warmth, dialog, the ability to show support to children, as well as to communicate using inductive reasoning to correct maladaptive behaviors of children (i.e., strategies of the acceptance/implication dimension), appears as the most appropriate strategy to achieve greater personal and social adjustment of children (Garcia and Gracia, 2010; Garcia and Serra, 2019; Garcia et al., 2020b, 2021; Martinez-Escudero et al., 2020; Queiroz et al., 2020).

In addition, the main findings from this study differ from those found in classic studies, mainly carried out with the European-American middle-class families, in which positive parenting is authoritative (i.e., strictness and warmth) (Baumrind, 1966; Steinberg et al., 1989, 1992; Lamborn et al., 1991; Darling and Steinberg, 1993). In European-American families, adolescents from authoritative families are those who are related to greater positive health, including not only fewer behavioral problems but also greater adjustment and wellbeing (Lamborn et al., 1991). The parenting styles in which one dimension is characterized by lower levels (i.e., authoritarian and indulgent) are associated with mixed results, while only the best benefits are related to the combination of strictness and warmth (i.e., authoritative parenting) (Lamborn et al., 1991). On the one hand, adolescents from authoritative and authoritarian families (both shared greater strictness) do not differ in academic performance, drug use, and delinquent behaviors. On the other hand, no differences were found between authoritative and indulgent styles (both shared greater warmth) in psychosocial development, including social competence and self-confidence (Lamborn et al., 1991).

Contrary to the results of classic studies with European-American families, those obtained in the present study suggest that the so-called positive parenting seems not to be authoritative but rather indulgent. Adolescents and adult children of indulgent families benefit from the warmth dimension since they show the greatest scores in self and internalization of universalism values and the lowest in emotional vulnerability. Therefore, these results contradict those found in classic studies (Lamborn et al., 1991; Steinberg et al., 1992, 1994), in which the component of strictness within authoritative parenting is emphasized to help children to achieve greater positive health. By contrast, according to the present study, it seems that the greatest benefits in terms of positive health are provided by those parents who are involved, caring, attentive to their needs, and who use reasoning and dialog to help their children understand the consequences of their actions on themselves and others. The strictness component seems to be unnecessary or even harmful to positive health. Therefore, it is worth noting the importance of the cultural context insofar as the values of the culture in which one lives could be the key to understanding the different results obtained regarding the appropriateness of the parental socialization style (Pinquart and Kauser, 2018; Garcia et al., 2021). The so-called positive parenting or effective parenting could not be always authoritative in all cultural contexts.

In addition, the present study examines the relationship between the parenting styles (i.e., authoritative, indulgent, authoritarian, and neglectful) and self (family self-concept, self-esteem, and negative self-efficacy), universalism values, and emotional vulnerability in adolescents and adult children. The pattern between parenting styles and health is quite similar; the parenting based on warmth was related to greater scores compared to non-warmth parenting, while the greatest scores correspond to parenting based on warmth without strictness (the indulgent), although some differences across age groups in men and women participants were found, especially in the most detrimental parenting styles (i.e., the authoritative and the authoritarian).

Specifically, the age profile showed on family self-concept showed that the authoritarian style is associated with slightly lower scores than the neglectful style, both styles are equal in the middle-aged group, and, at the end of life, this middle-aged trend was also observed in women, but in men, the neglectful style was associated with better family self-concept than the authoritarian style. In women participants, the neglectful style is associated with low but relatively stable scores in all four age groups, whereas, in men participants, they tend to report lower scores even from young adulthood, once parental socialization has ended, until the end of life when they have lower scores even than those raised by authoritarian parents. Women participants present a better family self-concept than men participants, so it is possible that, with the same type of family (neglectful) characterized by low warmth, men participants are more affected especially when the family socialization process ends. In addition, the age profile for women on emotional vulnerability revealed the harmful consequences associated with authoritative parenting, which are related to similar scores as neglectful parenting in young adults and specially marked in the middle-age group, the authoritarian parenting related to relatively stable but lower scores, and the neglectful, which is especially harmful in old age. Women tend to have greater emotional vulnerability than men, but only when being raised in homes characterized by warmth and without strictness, they have the maximum protection against emotional vulnerability. The age profile for mens participants tends to be more similar across age groups with lower scores between warm parenting compared to non-warm parenting.

This study presents some strengths and limitations. First, the use of a two-dimensional model and four typologies of parental socialization provides a common theoretical framework with which to compare research results from different countries. In the present study, the categorization of families (i.e., authoritative, indulgent, authoritarian, and neglectful) is provided by the split procedure on each dimension (i.e., warmth and strictness). The use of the split procedure, a statistical technique to assign participants to the parenting styles based on the measures of the two parenting dimensions, is a strategy commonly used in studies from different parts of the world, for example, the United States (Lamborn et al., 1991; Steinberg et al., 1994; Milevsky, 2020) or Europe (Calafat et al., 2014; Climent-Galarza et al., 2022; Fuentes et al., 2022). Other parenting typologies different from the classical parenting styles have been examined based on clustering methods such as latent profile analysis (LPA) (Borden et al., 2014; Ayon et al., 2015; Huang et al., 2022). Second, a large and broad sample has been used. In addition, the relationship between socialization styles and personal and social adjustment variables in both adolescent and adult children (young, middle-aged, and older) is analyzed. In this way, we contribute to the current debate on the best socialization strategy, and, in addition, the results of this study allow us to test whether the differences in personal and social adjustment among adults are also theoretically predictable from parenting styles. As limitations, it should be considered that self-reported questionnaires were used, which were answered by adolescent and adult children, although there is evidence that social desirability is lower in children than in parents (Barry et al., 2008). It should be considered that participants are mainly from middle-class families, so future studies should examine families from other socioeconomic backgrounds (e.g., poor neighborhood). Moreover, this is a cross-sectional study, so no conclusion can be drawn about the impact of parenting styles in the long term. In addition, the results should be interpreted with some caution, without inferring causal relationships.

Finally, as a conclusion, it should be noted that the present study contributes to current research on the appropriateness of parental socialization styles (Fuentes et al., 2015; Lorence et al., 2019; Garcia et al., 2021). On the one hand, the results of this research coincide with several studies conducted mainly in European and Latin American countries (Martínez and Garcia, 2008; Garcia and Gracia, 2010; Martinez et al., 2020), suggesting that the indulgent style is the best parental socialization style. However, these results do not coincide with those found in classic studies, carried out mainly with samples of American and ethnic minority families from Anglo-Saxon countries (Baumrind, 1978; Darling and Steinberg, 1993; Lim and Lim, 2003). The cultural context in which socialization takes place seems to influence the relationship between parental socialization styles and the pattern of personal and social adjustment of children (Pinquart and Kauser, 2018; Garcia et al., 2021). Future research should analyze what is the appropriate parental strategy for the education and adequate development of children, considering families from different cultural and ethnic backgrounds, as well as the individual differences of the children.

## Data availability statement

The raw data supporting the conclusions of this article will be made available by the authors via email request to the corresponding author, without any undue reservation.

## Ethics statement

The studies involving human participants were reviewed and approved by College Research Ethics Committee (CREC) of Nottingham Trent University (protocol code no. 2017/90, May 2017). Written informed consent to participate in this study was provided by the participants’ legal guardian/next of kin.

## Author contributions

MA and FG organized the database and wrote sections of the manuscript. IP and MA performed the statistical analysis. IP and OG wrote the first draft of the manuscript. All authors contributed to conception and design of the study, manuscript revision, read, and approved the submitted version.
